# Chromosome behavior during meiosis in pollen mother cells from *Saccharum officinarum × Erianthus arundinaceus* F_1_ hybrids

**DOI:** 10.1186/s12870-021-02911-z

**Published:** 2021-03-16

**Authors:** Xueting Li, Fei Huang, Jin Chai, Qiusong Wang, Fan Yu, Yongji Huang, Jiayun Wu, Qinnan Wang, Liangnian Xu, Muqing Zhang, Zuhu Deng

**Affiliations:** 1 National Engineering Research Center for Sugarcane, Fujian Agriculture and Forestry University, Fuzhou, 350002 Fujian China; 2 Guangdong Key Laboratory of Sugarcane Improvement and Biorefinery, Guangdong Provincial Bioengineering Institute, Guangzhou, China; 3 Key Lab of Sugarcane Biology and Genetic Breeding, Ministry of Agriculture, Fujian Agriculture and Forestry University, Fuzhou, 350002 Fujian China; 4 State Key Laboratory for Protection and Utilization of Subtropical Agro-Bioresources, Guangxi University, Nanning, 530004 China

**Keywords:** Chromosome, Meiosis, Pollen, FISH, Cell genetics

## Abstract

**Background:**

In recent years, sugarcane has attracted increasing attention as an energy crop. Wild resources are widely used to improve the narrow genetic base of sugarcane. However, the infertility of F_1_ hybrids between *Saccharum officinarum* (*S. officinarum*) and *Erianthus arundinaceus* (*E. arundinaceus*) has hindered sugarcane breeding efforts. To discover the cause of this infertility, we studied the hybridization process from a cytological perspective.

**Results:**

We examined the meiotic process of pollen mother cells (PMCs) in three F_1_ hybrids between *S. officinarum* and *E. arundinaceus*. Cytological analysis showed that the male parents, Hainan 92–77 and Hainan 92–105, had normal meiosis. However, the meiosis process in F_1_ hybrids showed various abnormal phenomena, including lagging chromosomes, micronuclei, uneven segregation, chromosome bridges, and inability to form cell plates. Genomic in situ hybridization (GISH) showed unequal chromatin distribution during cell division. Interestingly, 96.70% of lagging chromosomes were from *E. arundinaceus*. Furthermore, fluorescence in situ hybridization (FISH) was performed using 45S rDNA and 5S rDNA as probes. Either 45S rDNA or 5S rDNA sites were lost during abnormal meiosis, and results of unequal chromosomal separation were also clearly observed in tetrads.

**Conclusions:**

Using cytogenetic analysis, a large number of meiotic abnormalities were observed in F_1_. GISH further confirmed that 96.70% of the lagging chromosomes were from *E. arundinaceus*. Chromosome loss was found by further investigation of repeat sequences. Our findings provide insight into sugarcane chromosome inheritance to aid innovation and utilization in sugarcane germplasm resources.

**Supplementary Information:**

The online version contains supplementary material available at 10.1186/s12870-021-02911-z.

## Background

As a typical C_4_ crop, sugarcane has a large biomass and accounts for 75% of sugar production worldwide [1, 2]. *Saccharum officinarum* (2n = 8x = 80), which belongs to *Saccharum*, was the important source of sugar genes in cultivars. Modern sugarcane cultivars are mainly derived from interspecific hybridization between sugarcane varieties or hybrids, leading to an increasingly narrow genetic background [3, 4]. To broaden the genetic base of sugarcane and improve its heterozygosity, sugarcane breeders use wild sugarcane-related genera such as *Erianthus*, *Sclerostachya* (Hack) *A. Camus*, *Narenga* Bor, and *Miscanthus* as a germplasm resource for inbreeding [5].

*Erianthus arundinaceus* (2n = 4x = 40 or 2n = 6x = 60) has favorable characteristics, such as resistance to insects, drought, and disease [6–8]. *E. arundinaceus* has a high tillering ability, strong growth and good amenability to ratooning [9, 10]. As a robust wild genetic resource, *E. arundinaceus* is frequently used in sugarcane breeding programs worldwide. This species is an important wild sugarcane germplasm resource in the *Saccharum* complex and was crossed with sugarcane as early as 1885 [6]; around the same time that *S. spontaneum* began to be used for hybridization. Although F_1_ hybrids were successfully obtained in 1931, exploration of these F_1_ hybrids has been very slow, mainly due to the high degree of male sterility [7]. After decades of effort, sugarcane breeders successfully obtained a true BC_1_ generation of *Saccharum* hybrids and *E. arundinaceus* at the Hainan Sugarcane Breeding Station in 2001. The F_1_ chromosome inheritance pattern of *S. officinarum* × *E. arundinaceus* hybrids was n + n [8], and the F_1_ clones were male sterile. When F_1_ was used as a female parent to cross with *Saccharum* spp., the chromosome inheritance pattern seemed to be 2n + n [11]. However, there have been few reports on the mechanisms responsible for the high pollen infertility of F_1_ or the BC_1_ chromosome inheritance pattern (2n + n). As such, the chromosome behavior of hybrid progeny from sugarcane and *E. arundinaceus* during meiosis remains unclear.

Fluorescence in situ hybridization (FISH) generally involves using genomic DNA or a portion of the genomic DNA, such as repeat sequences and single-copy gene sequences, as probes [12]. The conserved, repetitive sequences in 45S rDNA and 5S rDNA are widely used to analyze plants’ evolutionary origin, identify chromosomes, and chromosome ploidy [13–15]. Genomic probes are widely used to discriminate chromosomes from two or more allopolyploid species and to distinguish the formation and evolution of different sources of polyploid species arising from chromosomal translocation [16], chromosomal loss [17], gene insertion [18], or chromosome-derived changes [19].

To analyze the chromosome behavior of pollen mother cells (PMCs) and cell division during meiosis, we performed cytological analyses of male *E. arundinaceus* (Hainan 92–77 and Hainan 92–105) and three F_1_ hybrids (Yacheng 96–40, Yacheng 96–66, and Yacheng 95–41). The FISH probes 45S rDNA and 5S rDNA were applied to investigate chromosomal behavior. The results from this study can provide a basis for the utilization of *E. arundinaceus* in sugarcane breeding programs.

## Materials and methods

### Plant material

Yacheng 96–40 and Yacheng 95–41 with significantly different phenotypes are F_1_ progeny from a cross of Badila (*S. officinarum* ♀) and Hainan 92–77 (*E. arundinaceus* ♂). Yacheng 95–41 had 28 chromosomes of *E. arundinaceus*, while Yacheng 96–40 had 29 chromosomes of *E. arundinaceus* [20]. Yacheng 96–66 resulted from a cross of Badila (*S. officinarum* ♀) and Hainan 92–105 (*E. arundinaceus* ♂), and the three progenies have different color of stem (Fig. S1). Yacheng 01–134 is the BC_1_ progeny from a cross of Yacheng 96–40 (♀) and ROC20 (*Saccharum* hybrids ♂). The plant materials were provided and preserved at the Hainan Sugarcane Breeding Station, China.

### Methods

#### Sampling and preservation

At 8:00–10:00 am, male buds in meiosis I where the anthers had not yet yellowed were fixed with Farmer’s fixative (ethanol: acetic acid = 3:1) for 24 h at 4 °C. Buds in the meiosis stage were confirmed by microscopic inspection and stored in 70% ethanol.

#### Staging of flower buds using acetocarmine staining

Two to three fixed buds were placed on a slide, and 50 μl of Farmer’s fixative was added. The anthers were squeezed lightly with dissection needles to release the meiotic cells, and impurities were then removed. Twenty microliters of acetocarmine solution (Solarbio, China, G1390–100 mL) was then added to stain the chromosomes. A cover slip was then placed on top and sealed with rubber cement.

#### FISH slide preparation

The collected staged anthers were washed with 75 mM KCl for 5 min in a 200 μL tube and twice with 10 mM citrate buffer (pH = 4.5) for 5 min. Then, 20 μL of 8% cellulase “ONOZUKA” R-10 (Yakult, Japan, MX7352), 20 μL of 2% pectinase (Sangon Biotech, China, A605099), and 20 μL of 1% pectolase Y-23 (Yakult, Japan, MX7354) were added, mixed gently subjected to enzymatic hydrolysis at 37 °C for 90 min. Finally, the samples were treated twice for 5 min with 10 mM citrate buffer at 4 °C, followed by precooled Farmer’s fixative, and stored at 4 °C.

#### Pollen viability assay

The mature anthers of Hainan 92–77, F_1_, and BC_1_ were collected. Two anthers were taken and placed on the slide, and a drop of ddH_2_O was added, and anthers were crushed with tweezers to release pollen grains. Then, 20 μL of I_2_-KI (1%) solution was added to cover the slide slowly, then pollen grains were checked with a microscope and photographed. Three slides were observed, and ten photos were taken from each slide. The dark-colored pollen were considered viable, whereas the light pollen were estimated to have poor viability.

#### Genomic in situ hybridization (GISH)

Buds after enzymolysis were quickly crushed with tweezers. PMCs in the buds were gently squeezed out to remove impurities, and 5 μL of the suspension was deposited onto a well-cleaned slide. Before the liquid dried, a drop of Farmer’s fixative was quickly added to the slide to spread the pollen evenly on the slide.

Genomic DNA was extracted using the CTAB method [21]. We used a nick translation kit (Roche, Switzerland, 10,976,776,001) to label the probes. The genomic DNA of *E. arundinaceus* (Hainan 92–77 genomic DNA) was labeled with digoxigenin-11-dUTP (Roche, Switzerland, 11,093,088,910). Bio16-dUTP (Roche, Switzerland, 11,093,070,910) was used to label the genomic DNA of *S. officinarum* (Badila genomic DNA). Hybridization solution containing the two DNA probes was prepared and dropped onto the slide, and then hybridization proceeded overnight in a humid chamber at 37 °C. The slides were washed in 2× SSC for 10 min at 42 °C, followed by washing with 2× SSC and 4× SSC/Tween for 5 min each at room temperature. To detect the signals from the digoxigenin and biotin probes, the slides were incubated with anti-digoxigenin-fluorescein (Roche, Switzerland, 11,207,741,910) and rhodamine antibodies (Vector, USA, A-2005) for 1 h at 37 °C and then washed three times with 4× SSC/Tween for 8 min at 37 °C. Antifade mounting medium with 4′-6-diamidino-2-phenylindole (DAPI, Vector, USA, H-1200) was used for counterstaining. Fluorescence imaging was performed using an AxioScope A1 imaging microscope and processed by AxioVision software.

## Results

### Pollen vitality test

**Fig. 1:**
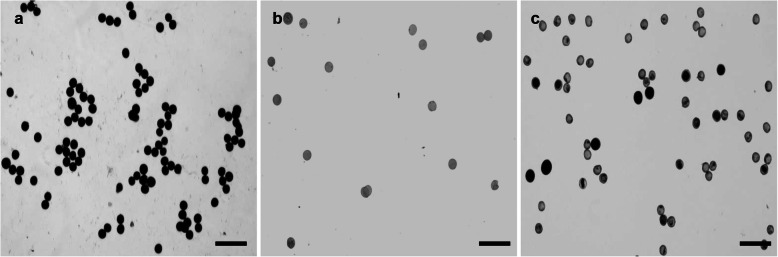
Iodine staining of pollen from Hainan 92–77 (*E. arundinaceus*), F_1_, and BC_1_. **a**: Paternal pollen grains. **b**: F_1_ pollen grains. **c**: BC_1_ pollen grains. Dark staining indicates viability, whereas light gray indicates no viability. Scale bars = 200 μm

I_2_-KI (1%) was used to stain mature pollen grains to detect the viability of F_1_ (Yacheng 96–40) pollen. The dark- and light-colored were considered viable and inviable, respectively. The fertility rate for paternal pollen (Hainan 92–77) grains was 98.39% (488/496) (Fig. 1a), whereas the F_1_ pollen grains were completely sterile (Fig. 1b). The pollen fertility of BC_1_ (Yacheng 01–134) recovered to 21.24% (452/2128) (Fig. 1c).

### Meiotic chromosome behavior of PMCs in *E. arundinaceus* and F_1_ hybrids

PMCs were stained with magenta acetate. Cells observed during meiosis were photographed, and all photographs taken were classified and counted; details are as follows.

**Fig. 2:**
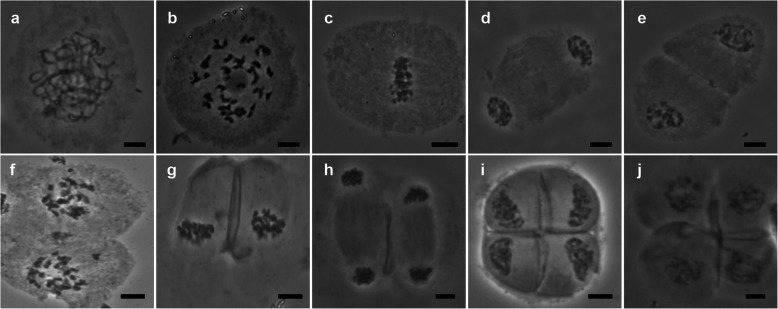
Hainan 92–77 showed normal meiotic behavior. **a**: Pachytene. **b**: Diakinesis. **c**: Metaphase I. **d**: Anaphase I. **e**: Telophase I. **f**: Dyad. **g**: Metaphase II. **h**: Anaphase II. **i**: Telophase II. **j**: Tetrad. Scale bars = 10 μm

PMC meiosis of fertile male parents Hainan 92–77 (Fig. 2a-j) and Hainan 92–105 (Fig. S2) exhibited normal division.

In the male parent, no abnormal behavior was observed in any period (Fig. 2a-j). However, three F_1_ individuals showed multiple abnormal behaviors during meiosis; Yacheng 96–40 (48.11%), Yacheng 95–41 (44.65%), and Yacheng 96–66 (46.44%) exhibited abnormalities, especially in anaphase I, in which more than 60% of the abnormalities occurred (Table 1).

**Table 1:** Observation and statistics of meiosis behavior of three F_1_(Yacheng 96–40, Yacheng 95–41 and Yacheng 96–66)

Name	Cells	Metaphase I	Anaphase I	Telophase I	Metaphase II	Anaphase II	Telophase II	Tetrad	Total
Yacheng 96–40	Total number of cells observed	218	244	88	187	125	92	158	1112
	Abnormal	96	163	23	75	94	26	58	535
	Percentage	44.04%	66.80%	26.14%	40.11%	75.20%	28.26%	36.71%	48.11%
Yacheng 95–41	Total number of cells observed	331	170	23	9	4	7	7	551
	Abnormal	124	108	6	3	2	1	2	246
	Percentage	37.46%	63.53%	26.09%	33.33%	50.00%	14.29%	28.57%	44.65%
Yacheng 96–66	Total number of cells observed	348	195	26	42	27	12	24	674
	Abnormal	145	116	4	19	18	3	8	313
	Percentage	41.67%	59.49%	15.38%	45.24%	66.67%	25.00%	33.33%	46.44%

“Abnormal” here encompasses all the observed abnormalities within each meiotic stage

**Fig. 3:**
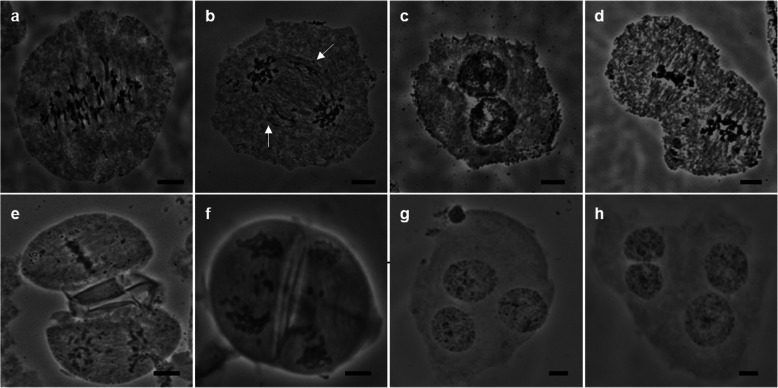
Typical abnormal phenomena observed in Yacheng 95–41 F_1_ meiosis processes. **a**: metaphase I, not synchronized. **b**: anaphase I, the white arrow points to the lagging chromosome. **c**: telophase I, dikaryocyte. **d**: metaphase II, no cell plate formed. **e**, **f**: anaphase II, chromosome division was not synchronized. **g**, **h**: Multinucleated cell, no cell plate formed. Scale bars = 10 μm

For example, several chromosomes were not synchronized (neatly arranged on the cell plate) in metaphase I (Fig. 3a). In anaphase I, lagging chromosomes were present (Fig. 3b). The cell plate did not form completely in telophase I (Fig. 3c), resulting in double nuclei at metaphase II (Fig. 3d). Similarly, chromosome separation was not synchronized in anaphase II (Fig. 3e), and lagging chromosomes were observed (Fig. 3f). Multiple nuclei were observed in telophase II (Fig. 3g, h). Yacheng 96–66 and Yacheng 96–40 had similar characteristics during meiosis (Fig. S3 and S4). In summary, our results suggested that F_1_ hybrids of Badila and *E. arundinaceus* exhibited abnormalities in meiosis, which might be a critical factor associated with pollen-related infertility.

### Analysis of GISH results

**Fig. 4:**
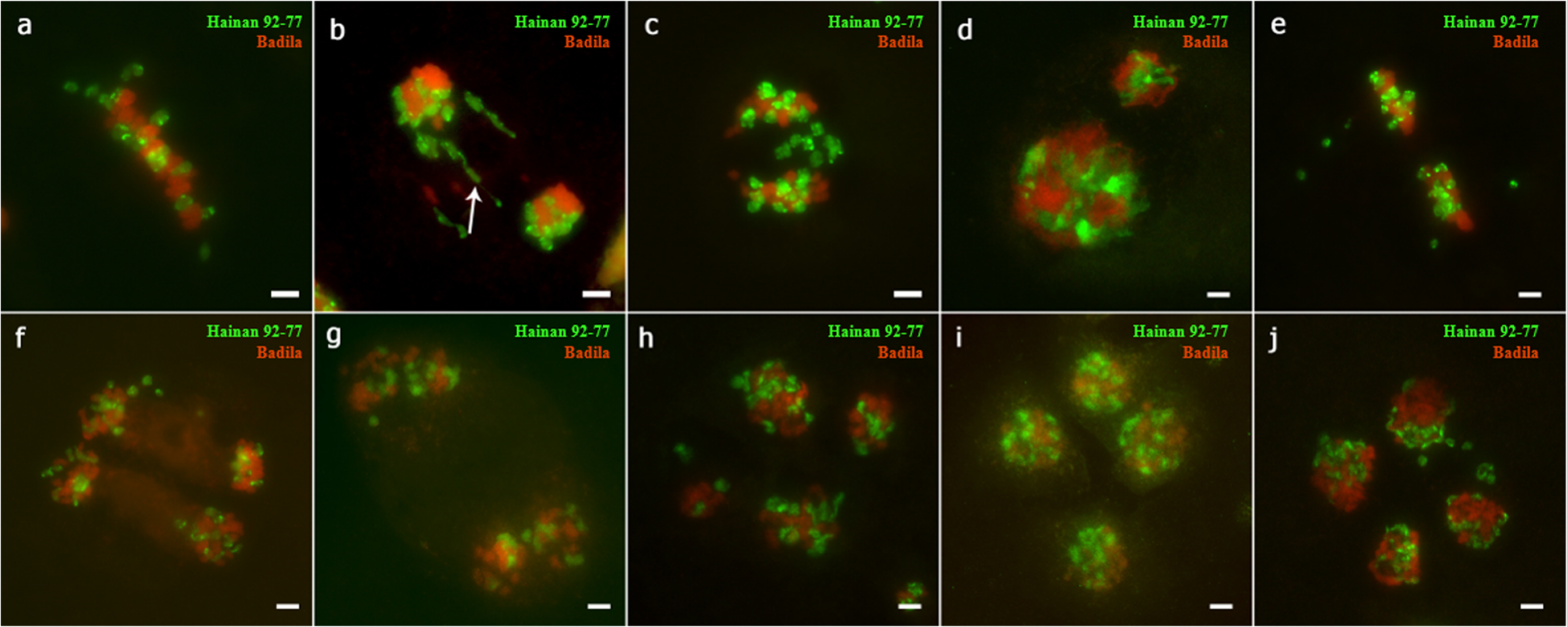
GISH of PMCs in F_1_ during abnormal meiosis. **a**: Lagging chromosome in metaphase I. **b**: The arrow points to the chromosome bridge. **c**: Lagging chromosome in anaphase I. **d**: Dikaryocyte. **e**: Lagging chromosome in telophase II. **f**: Lagging chromosome in telophase II. **g**: No cell plate formed in telophase II. **h**: Unsynchronized separation of tetrads. **i**: Unequal segregation in tetrads. **j**: Lagging chromosome and micronuclei in tetrads. Scale bars = 5 μm

The normal process of meiosis from prophase I to tetrad formation was followed by GISH (Fig. S5). Interestingly, we observed some bivalents between *S. officinarum* and *E. arundinaceus* during diakinesis (Fig. S5b). However, many abnormal phenomena were also observed. In metaphase I, when chromosomes were arranged on the cell plate, all lagging chromosomes belonged to *E. arundinaceus* (Fig. 4a). There were multiple chromosome bridges (Fig. 4b). We also detected lagging chromosomes from *E. arundinaceus* in anaphase I (Fig. 4c). During prophase II, the cell size differed markedly due to separation lag or unequal splitting (Fig. 4d). In metaphase II, *E. arundinaceus* chromosomes lagged (Fig. 4e). In anaphase II, a lagging chromosome was present (Fig. 4f). In telophase II, no cell plate formed (Fig. 4g). Unsynchronized separation (Fig. 4h), and unequal segregation of chromosomes led to a large nuclear size (Fig. 4i). The quantity of chromatin differed substantially among tetrads, and there were frequent instances of cells with chromosome lag and micronuclei (Fig. 4j).

### Analysis of FISH results

**Fig. 5:**
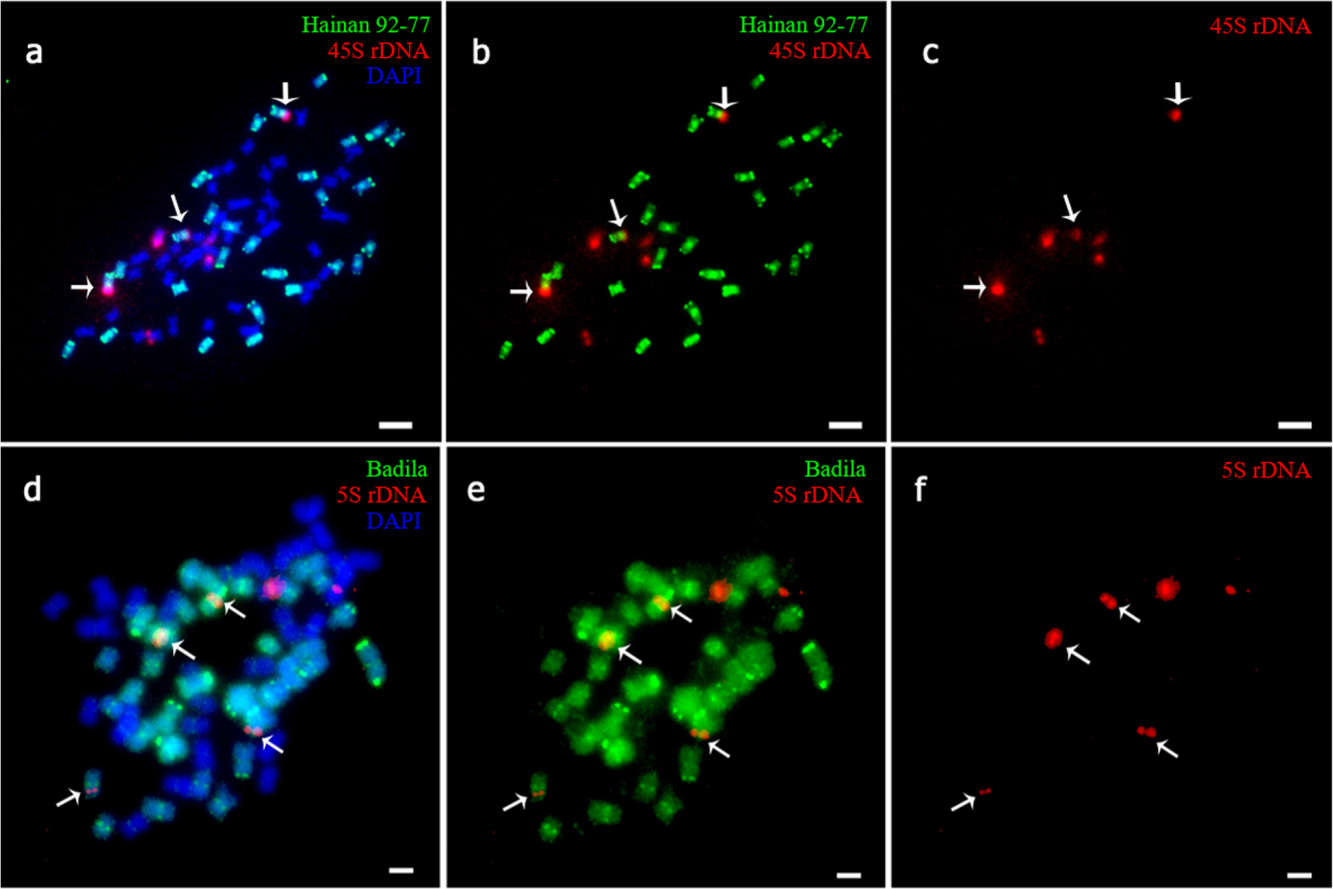
FISH mapping of 45S rDNA and 5S rDNA sites on somatic cell chromosomes of Yacheng 95–41. a-c: Somatic cell karyotype of Yacheng 95–41; white arrows indicate the three 45S sites in Hainan 92–77 (*E. arundinaceus*). d-f: Somatic cell karyotype of Yacheng 95–41; white arrows indicate the four 5S sites in Badila (*S. officinarum*). Scale bars = 5 μm

According to previous reports [20, 22], 45S and 5S rDNA had one locus per set of chromosomes in Badila and *E. arundinaceus*. At mitosis, Hainan 92–77 had six 45S and six 5S rDNA sites (Fig. Sa, b), and Badila had eight 45S and eight 5S rDNA sites at mitosis (Fig. Sc, d). F_1_ somatic cells had seven 45S rDNA sites (Fig. 5a-c), including four from *S. officinarum* and three from *E. arundinaceus*. The chromosome composition of F_1_ cells was 2n = n (*S. officinarum*) + n (*E. arundinaceus*). Meanwhile, there were six 5S rDNA sites in somatic cells, of which four and two sites were from *S. officinarum* and *E. arundinaceus*, respectively (Fig. 5d-f).

**Fig. 6:**
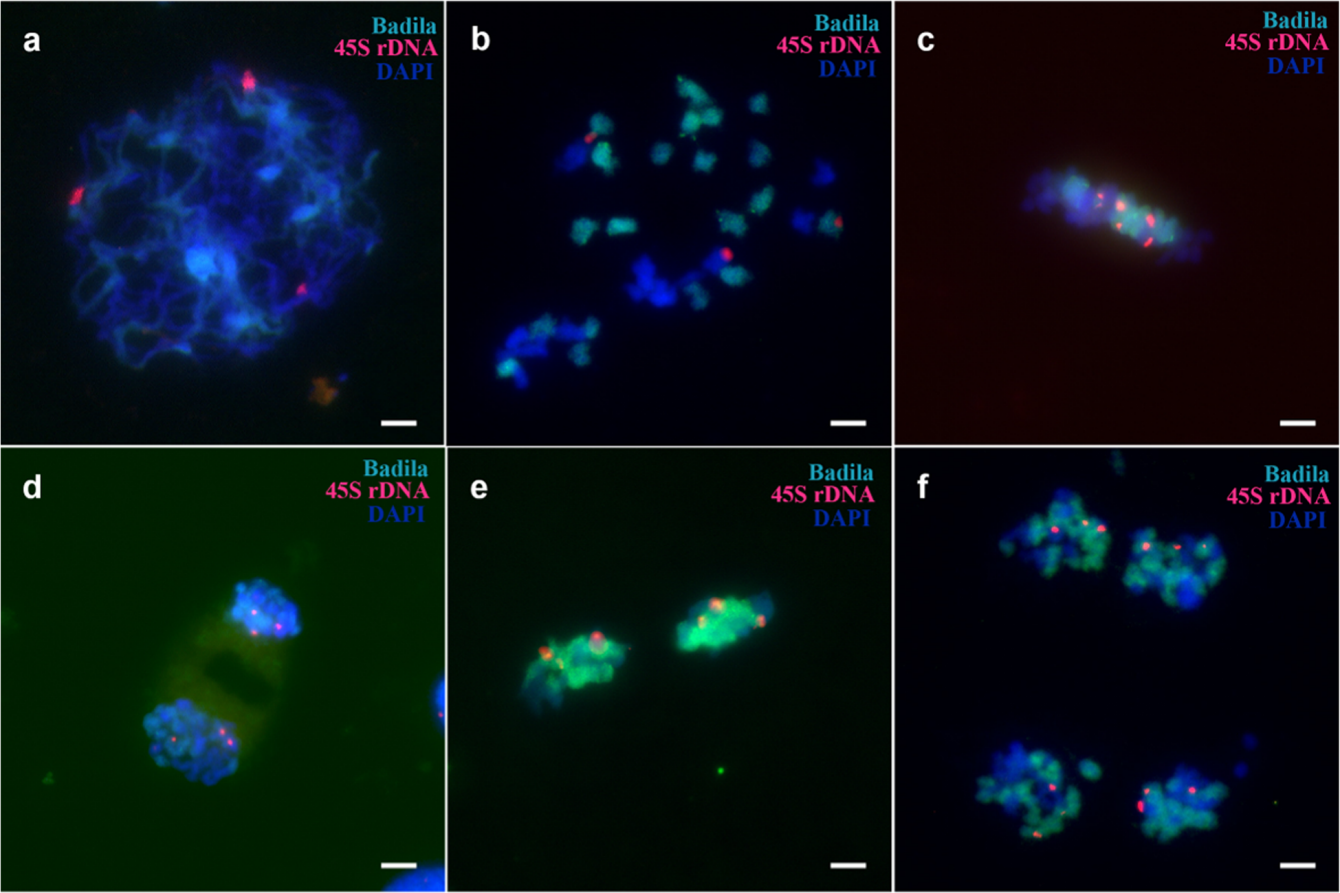
FISH mapping of 45S rDNA sites on chromosomes of Yacheng 95–41 during pollen mother cell (PMC) meiosis. Green foci are the 45S rDNA sites, and the chromosomes are stained with DAPI. **a**: Pachytene, **b**: Diakinesis, **c**: Metaphase I, **d**: Anaphase I, **e**: Metaphase II, **f**: Tetrad. Scale bars = 5 μm

**Fig. 7:**
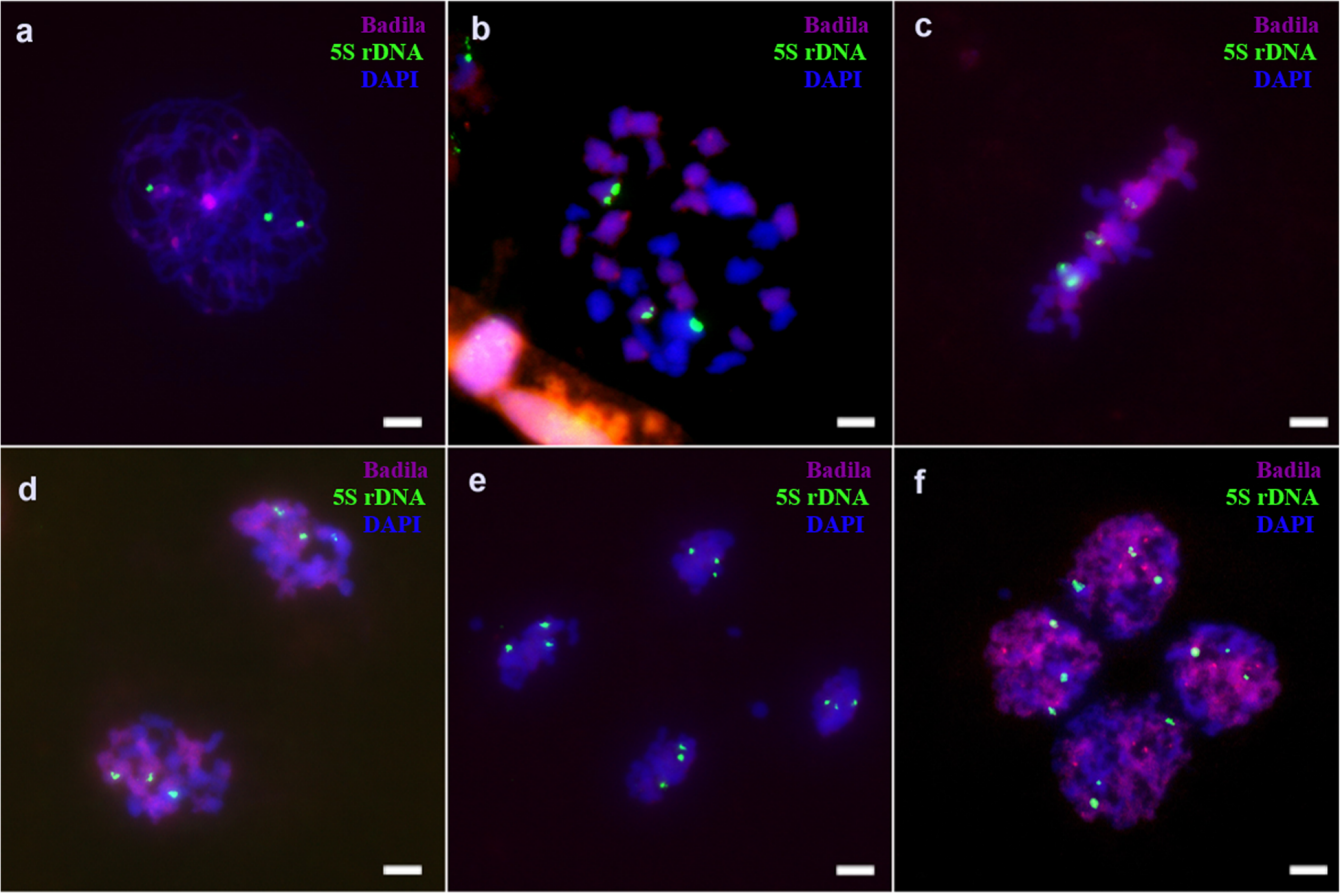
FISH mapping of 5S rDNA sites on chromosomes of Yacheng 95–41 during pollen mother cell (PMC) meiosis. The green foci are the 5S rDNA sites. Chromosomes are stained with DAPI. **a**: Pachytene, **b**: Diakinesis, **c**: Metaphase I, **d**: Anaphase I, **e**: Telophase I, **f**: Tetrad. Scale bars = 5 μm

However, during F_1_ pollen meiosis, only six 45S rDNA sites appeared, fewer than the seven sites seen in Yacheng 95–41 somatic cells (Fig. 6). Additionally, the 45S rDNA locus could be normally distributed in each cell during meiosis. During meiosis I, 5S rDNA was always detected at six sites (Fig. 7). Our results revealed that one 45S rDNA locus was lost during meiosis II (Fig. S7), whereas the number of 5S rDNA sites in each cell varied during meiosis (Fig. S8). Anomalies were found in the tetrad period (Fig. S9).

## Discussion

Sterility is a key challenge that hinders the crossbreeding and utilization of *E. arundinaceus*. The low fertility of hybrids also affects distant hybridization in other species, such as barley [23, 24], wheat [25], and potato [26]. Many previous studies have used cytology to understand the phenomena that contribute to this low fertility. Bremer hypothesized that offspring produced by intergeneric hybridization will tend to exhibit unbalanced chromosome distribution, termed “unbalanced hybridization” [27]. As an allopolyploid plant, sugarcane has been thought to be able to accommodate foreign gene resources relatively easily and thus allow generation of interspecific and intergeneric hybrids, particularly those involving hybridization with *E. arundinaceus.* Unbalanced hybridization also occurred in hybrids between sugarcane and *E. arundinaceus* [27]. The high sterility of PMC in F_1_ was detected by the germination rate of hybrid spikes, and fewer *E. arundinaceus* chromosomes were found in F_1_ plants [28]. This report confirmed the phenomenon of chromosome loss during meiosis in this study. Only 29 *E. arundinaceus* chromosomes were detected using GISH, and one chromosome was missing in hybrids [29]. Due to F_1_ male sterility, F_1_ could only be used as the female parent, the offspring material could be obtained through backcrossing, and the chromosome transmission was 2n + n or more than 2n + n, which also made the BC_1_ material richer in a variety of resistances and vitalities. *E. arundinaceus* chromosomes were also lost (28 ~ 29) in five hybrids [30]. When these hybrids were crossed with commercial varieties as female parents, the pattern of “2n + n” or more than “2n + n” was seen in BC_1_ hybrids, and 23 ~ 36 *E. arundinaceus* chromosomes were present in these plants. The fertility of BC_1_ material was restored, and the restoration of the fertility of the offspring helped obtain “noble” hybrids with the advantages of high sugar content, high disease resistance, and strong tillers [30]. In general, low fertility and unbalanced hybridization in offspring were most likely related to abnormal meiosis processes.

In this study, various abnormal phenomena, such as lagging chromosomes, asynchronism, micronuclei, and inability to form cell plates, were observed during pollen development in F_1_. In particular, in anaphase of meiosis I and II, the abnormal cell population reached approximately 70%. Similar results were seen for other species. For example, the progeny of wheat × rye and F_1_ of *Triticum timopheevii* × hexaploid wild oat had abnormal chromosome bridges, micronuclei, and other abnormal behaviors [31, 32], which might be reasons for low fertility. Chromosome pairing between sugarcane and *E. arundinaceus* showed diakinesis and coincided with chromosome translocation and exchange in BC_1_ [20]. Chromosome arrangement at the mitotic metaphase in cotton hybrids followed regular spatial separation of the genome [33]. Distant hybridization causes chromosome rearrangement [34]. Competition between genomes; differences in the timing of centromere division, chromosome number, and cell cycle length between parents; and interactions between the nucleus and cytoplasm might be the causes of lagging chromosomes [35–37]. Our results revealed that chromosomes from *S. officinarum* tended to cluster together. Furthermore, invasion by many exogenous chromosomes might have contributed to the phenomenon of lag. Approximately 3x chromosomes from *E. arundinaceus* were transmitted to F_1_ hybrids, which facilitated abnormal chromosome pairing. Hence, all lagging chromosomes were from *E. arundinaceus*. Micronucleus formation was caused by chromosome lag that prevented chromosomes from entering newly formed cells. Many cells did not form a cell plate during telophase, leading to the emergence of cells with two or more nuclei. This phenomenon was similar to that of abnormal meiosis observed in rice pollen (male sterile line of Zhenshan 97A and its maintaining line Zhenshan 97B) [38]; The nuclear membrane and cell plate disintegrated rapidly during meiosis I, and the tapetum cells also disintegrated rapidly. The variation in centromeric histone H3 acts as a barrier to species hybridization, which may be the reason for the elimination of male parent chromosomes in the process of hybridization [39]. The spatial segregation of chromosomes and the structural recombination of paternal chromosomes resulted in the formation of micronuclei and haploids [40]. Several factors that influence cell plate formation were outlined, including temperature, molecular regulation, and callose deposition [41]. However, the detailed mechanisms underlying these observations in sugarcane are unclear.

Using 45S rDNA as a probe, we observed only three 45S loci in diakinesis, which did not exclude the possibility of trivalent complexes. Before chromosome doubling, six 45S rDNA sites were present during meiosis. The causes and mechanisms of 45S rDNA locus loss require further study. A *Tragopogon micelles* gene was lost during chromosome recombination in neo-allotetraploid hybrids, which might have been caused by abnormal chromosome pairing [42]. Additionally, we used 5S rDNA as a probe for chromosome tracking and detected two obviously abnormal types, with 2, 2, 4, and 4 loci and 2, 2, 3, and 5 loci in the four daughter cells at the tetrad stage. This result indicated that unequal segregation of the chromosomes with 5S rDNA occurred during meiosis. The unequal segregation was likely caused by lagging *E. arundinaceus* chromosomes.

## Conclusion

F_1_ hybrids of sugarcane and *E. arundinaceus* showed high rates of sterility. These hybrids also exhibited multiple abnormal phenomena during meiosis, such as chromosome lag, asynchronous chromosome separation, presence of micronuclei, and inability to form a cell plate. Furthermore, in 96.70% (176/182) of cells with lagging chromosomes, the lagging chromosomes were from *E. arundinaceus*. The location of the 45S rDNA and 5S rDNA indicated that unequal division and loss of chromosomes were present in F_1_ PMCs. The results revealed that sterility was caused by disturbances in meiosis, unequal segregation, and chromosome damage. This study provided the first cytological evidence to show abnormal chromosome behavior during meiosis of PMCs from these hybrids and directly suggested a mechanistic basis for PMC sterility in F_1_ hybrids. These results will lay a foundation for further related research on sugarcane intergeneric germplasm for sugarcane breeding.

## Supplementary Information

**Additional file 1: Fig. S1.** Plant phenotype of F_1_. a: Yacheng 96–40 stem showing mostly red pigmentation. b: Yacheng 95–41 stem showing green pigmentation. c: Yacheng 96–66 stem showing light yellow pigment.

**Additional file 2: Fig. S2.** Hainan 92–105 shows normal meiotic behavior. a: Diakinesis. b: Metaphase I. c and d: Anaphase I. e: Telophase I. f: Metaphase II. g: Anaphase II. h: Tetrad. Scale bars = 10 μm.

**Additional file 3: Fig. S3.** Abnormal meiosis processes in F_1_ (Yacheng 96–40). a: Lagging chromosome in metaphase I. b: Lagging chromosome in anaphase I. c, d and g: The cell plate did not form completely in telophase I, metaphase II and telophase II. d: The cell plate did not form completely. e: Asynchronous division in anaphase II. f: Lagging chromosome in anaphase II. h: Triad. Scale bars = 10 μm.

**Additional file 4: Fig. S4.** Abnormal meiosis processes in F_1_ (Yacheng 96–66). a: Chromosome was not synchronized in metaphase I. b: Lagging chromosome in anaphase I. c: Lagging chromosome telophase I. d: No new cell plates formed in metaphase II. e and f: Asynchronous division in anaphase II. g: Cell with four nuclei. h: Asynchronous division in telophase II. Scale bars = 10 μm.

**Additional file 5: Fig. S5.** GISH of F_1_ PMCs during normal meiosis. a: Pachytene. b: Diakinesis, white arrow refers to paired bivalents. c: Metaphase I. d: Anaphase I. e: Telophase I. f: Metaphase II. g: Anaphase II. h: Tetrad. Scale bars = 5 μm.

**Additional file 6: Fig. S.** FISH mapping results for 45S rDNA and 5S rDNA at somatic cell chromosomes for Hainan 92–77 and Badila. a and b: somatic cell of Hainan 92–77. c and d: somatic cell of Badila. Arrows point to 45S rDNA and 5S rDNA foci. Scale bars = 5 μm.

**Additional file 7: Fig. S7.** Abnormal meiosis processes in F_1_ by 45S rDNA FISH mapping results. a: 45S site in pachytene. b: 45S site in metaphase I. c:45S site in anaphase I, the square indicated the lagging chromosome with 45S rDNA. Scale bars = 5 μm.

**Additional file 8: Fig. S8.** Abnormal meiosis processes in F_1_ by 5S rDNA FISH mapping results. a: 5S site in anaphase I. b: 5S site in anaphase II. c: 5S rDNA site in tetrad. Scale bars = 5 μm.

**Additional file 9: Fig. S9.** Abnormal meiosis processes in F_1_ by 45S rDNA and 5S rDNA FISH mapping results. a and b: 45S rDNA and 5S rDNA site in tetrad. Scale bars = 5 μm.

## Data Availability

The datasets used and analysed during the current study are available from the corresponding author on reasonable request, the experimental materials have been approved for use by the Hainan Sugarcane Breeding Station, China. Follow institutional, national or international guidelines.

## Abbreviations

PMC: Pollen’s mother cell
GISH: Genomic in situ hybridization
FISH: Fluorescence in situ hybridization
CTAB: Cetyltrimethylammonium bromide
DAPI: 4′-6-diamidino-2-phenylindole
SSC: saline-sodium citrate

## References

[1] Racedo J, Gutierrez L, Perera MF, Ostengo S, Pardo EM, Cuenya MI, Welin B, Castagnaro AP (2016). Genome-wide association mapping of quantitative traits in a breeding population of sugarcane. BMC Plant Biol.

[2] Qian YLX, Yifeng Z, Youxiong Q (2013). Genetic diversity analysis of sugarcane parents in Chinese breeding Programmes using gSSR markers. Sci World J.

[3] Rajeswari S, Sekar S, Krishnamurthi M: Development of subclonal variants from interspecific hybrids of sugarcane. Biotechnology and Sustainable Agriculture 2006 and Beyond 2007:433.

[4] Karpagam E, Alarmelu S (2017). Morphological characterization and genetic diversity analysis of interspecific hybrids of sugarcane. Indian J Genet Pl Br.

[5] Amalraj VA, Balasundaram N (2006). On the taxonomy of the members of ‘*Saccharum* Complex’. Genet Resources Crop Evol.

[6] Li WF, Wang XY, Huang YK, Shan HL, Luo ZM, Ying XM, Zhang RY, Shen K, Yin J (2013). Screening sugarcane germplasm resistant to Sorghum mosaic virus. Crop Prot.

[7] He SC, Yang QH, Xiao FH, Zhang FC, He LL. Collection and description of basic germplasm of sugarcane (*Saccharum complex*) in China. Int Sugar J. 1999;101(1201):84–85,88–89,92–93.

[8] Berding N, Pendrigh RS (2009). Breeding implications of diversifying end uses of sugarcane. Int Sugar J.

[9] Bhat SR, Gill SS (1985). The implications of 2n egg gametes in nobilization and breeding of sugarcane. Euphytica.

[10] Zu-Hu D, Mu-Qing Z, Wei-Le L, Fu C, Chui-Ming Z, Yu-Chang LI, Li-Ping L, Yan-Quan L, Ru-Kai C. Analysis of disequilibrium hybridization in hybrid and backcross progenies of *Saccharum officinarum* x *Erianthus arundinaceus*. J Integr Agric 2010, 9(9):1271–7.

[11] Hermann SR, Aitken KS, Jackson PA, George AW, Piperidis N, Wei X, Kilian A, Detering F (2012). Evidence for second division restitution as the basis for 2n + n maternal chromosome transmission in a sugarcane cross. Euphytica.

[12] Kim C, Robertson JS, Paterson AH (2011). Inference of subgenomic origin of BACs in an interspecific hybrid sugarcane cultivar by overlapping oligonucleotide hybridizations. Genome.

[13] Fernando R, Marcelo G (2012). Distribution of 45S rDNA sites in chromosomes of plants: structural and evolutionary implications. BMC Evol Biol.

[14] de Melo NF, Guerra M (2003). Variability of the 5S and 45S rDNA sites in Passiflora L. species with distinct base chromosome numbers. Ann Bot.

[15] D'Hont A, Grivet L, Feldmann P, Glaszmann JC, Rao S, Berding N. Characterisation of the double genome structure of modern sugarcane cultivars (*Saccharum* spp.) by molecular cytogenetics. Mol Gen Genet. 1996;250(4):405–13. 10.1007/BF02174028 8602157

[16] Kopecky D, Martis M, Cihalikova J, Hribova E, Vrana J, Bartos J, Kopecka J, Cattonaro F, Stoces S, Novak P (2013). Flow sorting and sequencing meadow fescue chromosome 4F. Plant Physiol.

[17] Harper J, Armstead I, Thomas A, James C, Gasior D, Bisaga M, Roberts L, King I, King J (2011). Alien introgression in the grasses *Lolium perenne* (perennial ryegrass) and *Festuca pratensis* (meadow fescue): the development of seven monosomic substitution lines and their molecular and cytological characterization. Ann Bot.

[18] Moscone EA, Matzke MA, Matzke AJ (1996). The use of combined FISH/GISH in conjunction with DAPI counterstaining to identify chromosomes containing transgene inserts in amphidiploid tobacco. Chromosoma.

[19] Jacobsen E, De Jong JH, Kamstra SA, van den Berg PMMM, Ramanna MS. Genomic in situ hybridization (GISH) and RFLP analysis for the identification of alien chromosomes in the backcross progeny of potato (+) tomato fusion hybrids. Heredity. 1995;74(3):250–7.

[20] Wu J, Huang Y, Lin Y, Fu C, Liu S, Deng Z, Li Q, Huang Z, Chen R, Zhang M (2014). Unexpected inheritance pattern of *Erianthus arundinaceus* chromosomes in the intergeneric progeny between *Saccharum* spp. and *Erianthus arundinaceus*. Plos One.

[21] Porebski S, Bailey LG, Baum BR (1997). Modification of a CTAB DNA extraction protocol for plants containing high polysaccharide and polyphenol components. Plant Mol Biol Report.

[22] Li L, Arumuganathan K (2001). Physical mapping of 45S and 5S rDNA on maize metaphase and sorted chromosomes by FISH. Hereditas.

[23] Molnár-Láng M, Galiba G, Kovács G, Sutka J. Changes in the fertility and meiotic behaviour of barley (*Hordeum vulgare*) × wheat (*Triticum aestivum*) hybrids regenerated from tissue cultures. Genome. 1991;34(2):261–6.

[24] Kim NS, Fedak G, Han F, Cao W (2008). Cytogenetic analyses of intergeneric hybrids between barley and nine species of *Elymus*. Genome.

[25] Lukaszewski AJ (2010). Behavior of centromeres in Univalents and centric Misdivision in wheat. Cytogenet Genome Res.

[26] Panahandeh J (2019). Chromosome pairing in auto-allotetraploid (AAAB) interspecific hybrid potatoes. New Zeal J Crop Hort.

[27] Bremer G (1961). Problems in breeding and cytology of sugar cane - III. The cytological crossing research of sugar cane. Euphytica.

[28] Piperidis G, Christopher M, Carroll B, Berding N. Molecular contribution to selection of intergeneric hybrids between sugarcane and the wild species *Erianthus arundinaceus*. Genome. 2000;43(6):1033–7. 11195335

[29] D'Hont A, Rao PS, Feldmann P, Grivet L, Islam-Faridi N, Taylor P, Glaszmann JC. Identification and characterisation of sugarcane intergeneric hybrids *Saccharum officinarum* x *Erianthus arundinaceus*, with molecular markers and DNA in situ hybridization. Theor Appl Genet. 1995;91(2):320–6. 10.1007/BF00220894 24169780

[30] Pachakkil B, Terajima Y, Ohmido N, Ebina M, Irei S, Hayashi H, Takagi H. Cytogenetic and agronomic characterization of intergeneric hybrids between *Saccharum* spp. hybrid and *Erianthus arundinaceus*. Sci Rep. 2019;9(1):1748. 10.1038/s41598-018-38316-6 PMC6370852 30742000

[31] Silkova OG, Adonina IG, Krivosheina EA, Shchapova AI, Shumny VK (2013). Chromosome pairing in meiosis of partially fertile wheat/rye hybrids. Plant Reprod.

[32] An H, Hu M, Li P, Geng G, Zhang Q, Zhang SJPO (2015). Chromosomal behavior during meiosis in the progeny of *Triticum timopheevii* × Hexaploid wild oat.

[33] Han JL, Zhou BL, Shan WB, Yu L, Wu W, Wang K. A and D genomes spatial separation at somatic metaphase in tetraploid cotton: evidence for genomic disposition in a polyploid plant. Plant J. 2015;84(6)(-):1167–77. 10.1111/tpj.13074 26568399

[34] Wang XH, Yang QH, Li FS, He LL, He SC (2010). Characterization of the chromosomal transmission of Intergeneric hybrids of *Saccharum* spp. and *Erianthus fulvus* by genomic in situ hybridization. Crop Sci.

[35] Silkova OG, Peresmyslova EE, Shchapova AI, Shumnyĭ VK (2008). Genetic regulation of the centromere division in rye and wheat univalent chromosomes in dimonosomics during meiotic anaphase I. Genetika.

[36] Wang CJ, Dai SF, Zheng YL (2010). Formation of unreduced gametes is impeded by homologous. Euphytica.

[37] Lyusikov OM, Bel’ko NB, Shchet’ko IS, Gordei IA (2005). Construction of Rye-wheat amphidiploids with the cytoplasm of Rye—Secalotriticum (RRAABB, 2n= 42): meiosis characteristics in Rye-triticale F1Hybrids (RRABR, 5x= 35). Russ J Genet.

[38] Xia KF, Wang YQ, Xiu-Lin YE, Liang CY, Xin-Lan XU. Ca~(2+) Distribution in the Tapetum in a Genic-Cytoplasmic Male Sterile Line of Rice, Zhenshan 97A and Its Maintainer Line Zhenshan 97B. Acta Bot Yunnanica. 2005;27(4):413–8.

[39] Sanei M, Pickering R, Kumke K, Nasuda S, Houben A. Loss of centromeric histone H3 (CENH3) from centromeres precedes uniparental chromosome elimination in interspecific barley hybrids. Proc Natl Acad Sci U S A. 2011;108(33):13373–4. 10.1073/pnas.1103190108 PMC3158150 21746892

[40] Dorota Gernand TR, Varshney A, Rubtsova M, Prodanovic S, Brüss C, Kumlehn J, Matzk F, Houben A (2005). Uniparental chromosome elimination at mitosis and interphase in wheat and pearl millet crosses involves micronucleus formation, progressive Heterochromatinization, and DNA fragmentation. Plant Cell.

[41] De Storme N, Geelen D (2013). Cytokinesis in plant male meiosis. Plant Signal Behav.

[42] Chester M, Lipman MJ, Gallagher JP, Soltis PS, Soltis DE (2013). An assessment of karyotype restructuring in the neoallotetraploid *Tragopogon miscellus* (Asteraceae). Chromosome Res.

