# Supplementary figures and images for: Chromosome behavior during meiosis in pollen mother cells from Saccharum officinarum × Erianthus arundinaceus F1 hybrids
Source: BMC Plant Biol. 2021 Mar 16;21:139. doi: 10.1186/s12870-021-02911-z (PMC7968283; doi:10.1186/s12870-021-02911-z)

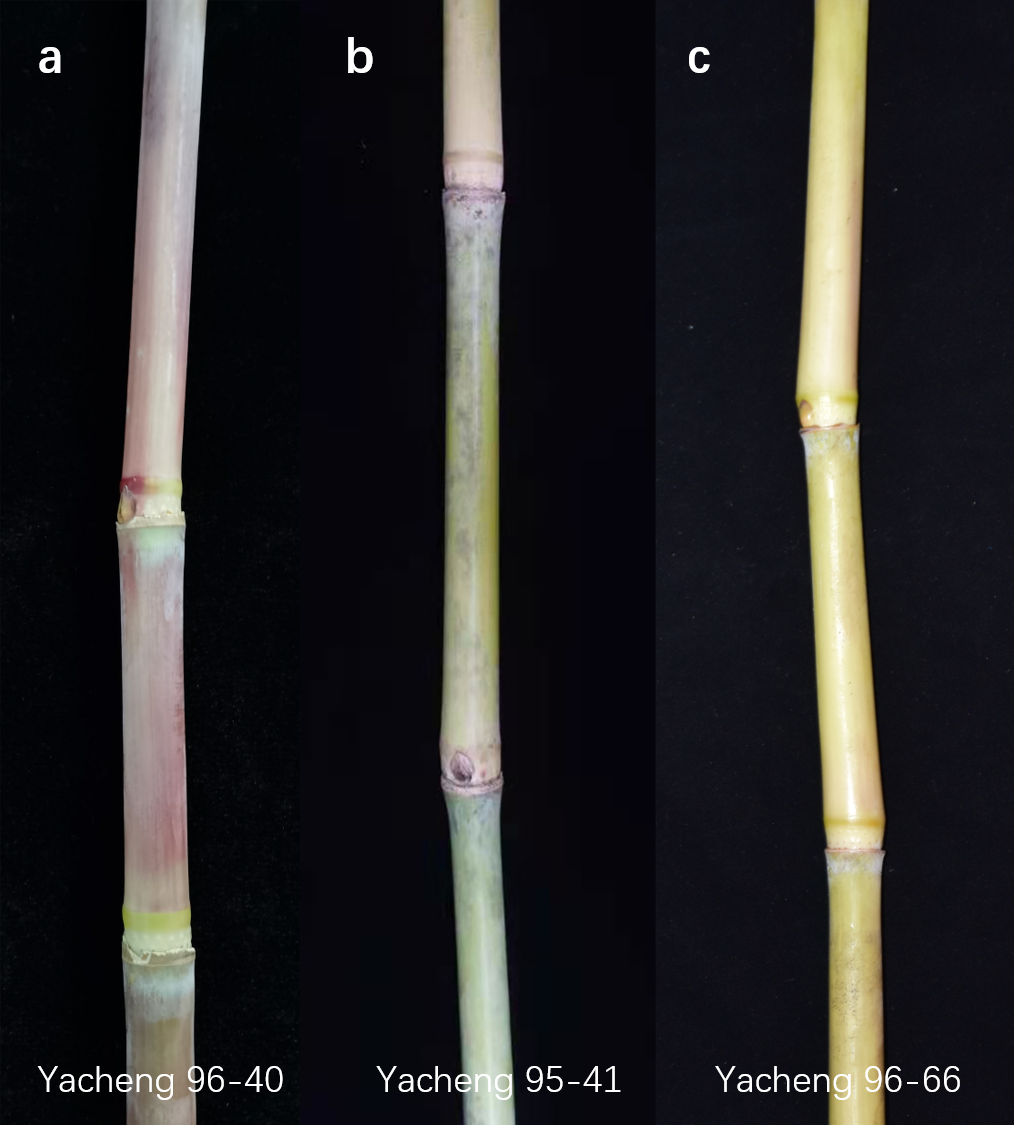

Supplement: Supplementary file 1 — Additional file 1: Fig. S1. Plant phenotype of F1. a: Yacheng 96–40 stem showing mostly red pigmentation. b: Yacheng 95–41 stem showing green pigmentation. c: Yacheng 96–66 stem showing light yellow pigment. [file 12870_2021_2911_MOESM1_ESM.tif]

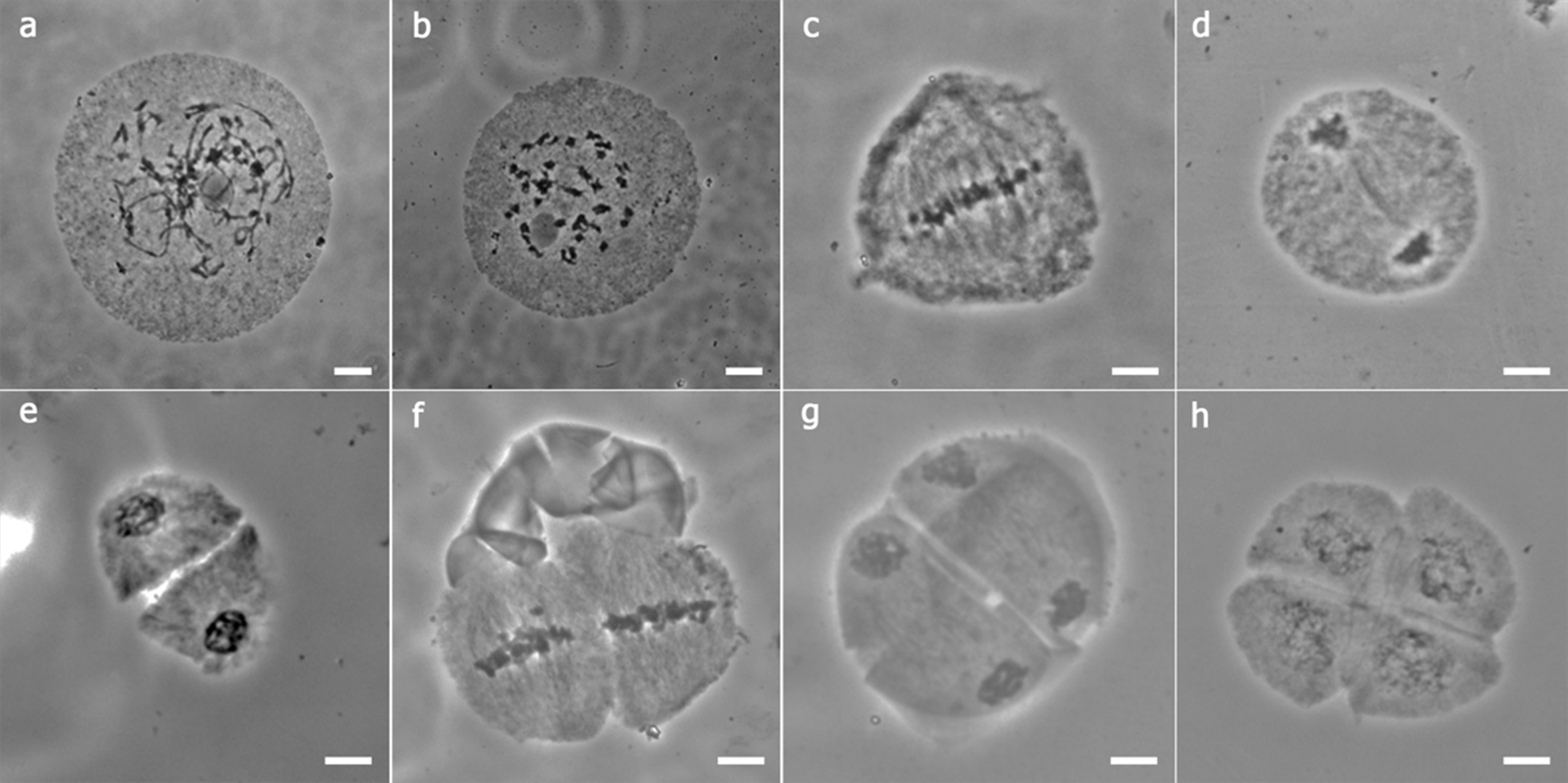

Supplement: Supplementary file 2 — Additional file 2: Fig. S2. Hainan 92–105 shows normal meiotic behavior. a: Diakinesis. b: Metaphase I. c and d: Anaphase I. e: Telophase I. f: Metaphase II. g: Anaphase II. h: Tetrad. Scale bars = 10 μm. [file 12870_2021_2911_MOESM2_ESM.tif]

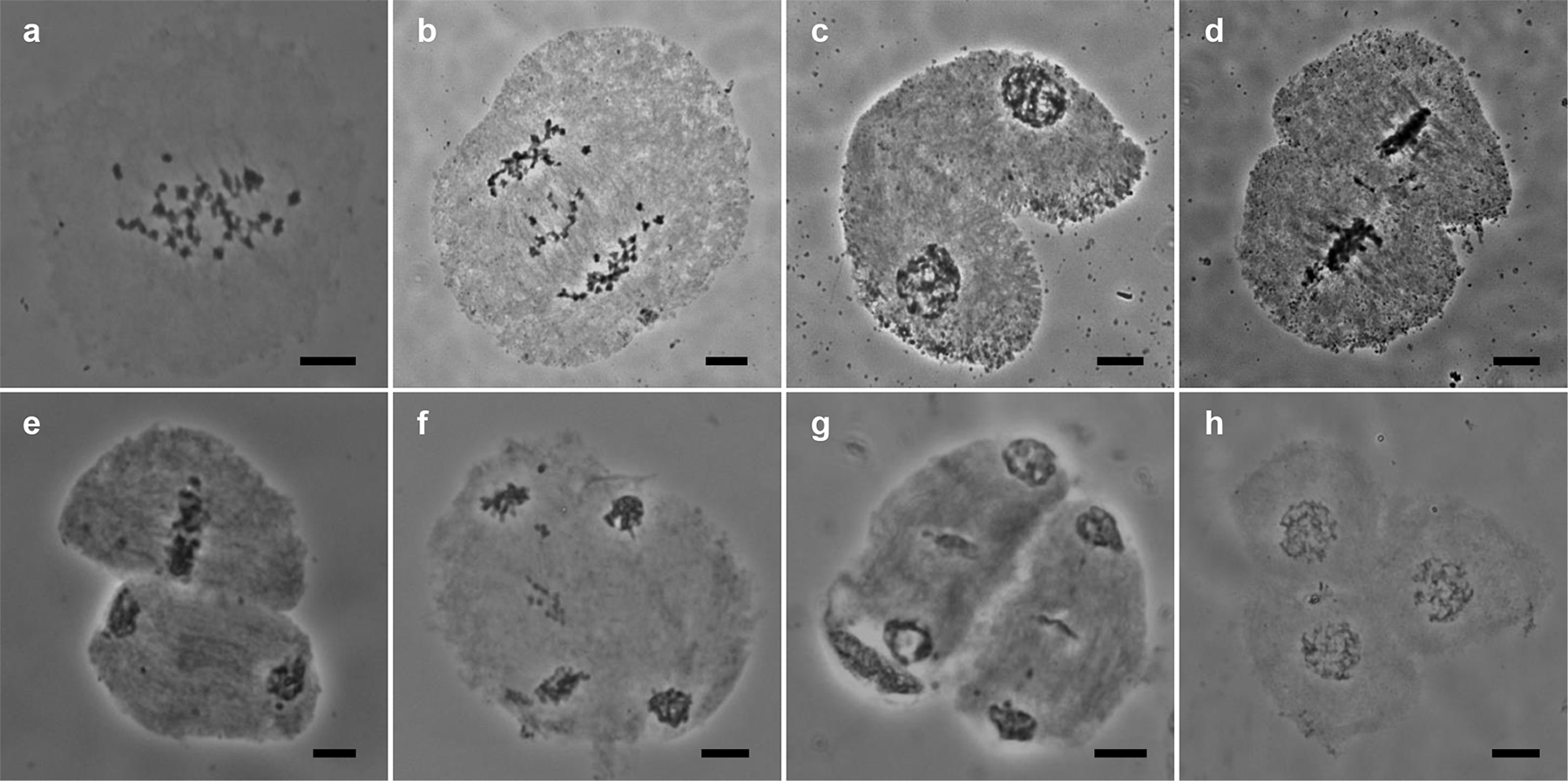

Supplement: Supplementary file 3 — Additional file 3: Fig. S3. Abnormal meiosis processes in F1 (Yacheng 96–40). a: Lagging chromosome in metaphase I. b: Lagging chromosome in anaphase I. c, d and g: The cell plate did not form completely in telophase I, metaphase II and telophase II. d: The cell plate did not form completely. e: Asynchronous division in anaphase II. f: Lagging chromosome in anaphase II. h: Triad. Scale bars = 10 μm. [file 12870_2021_2911_MOESM3_ESM.tif]

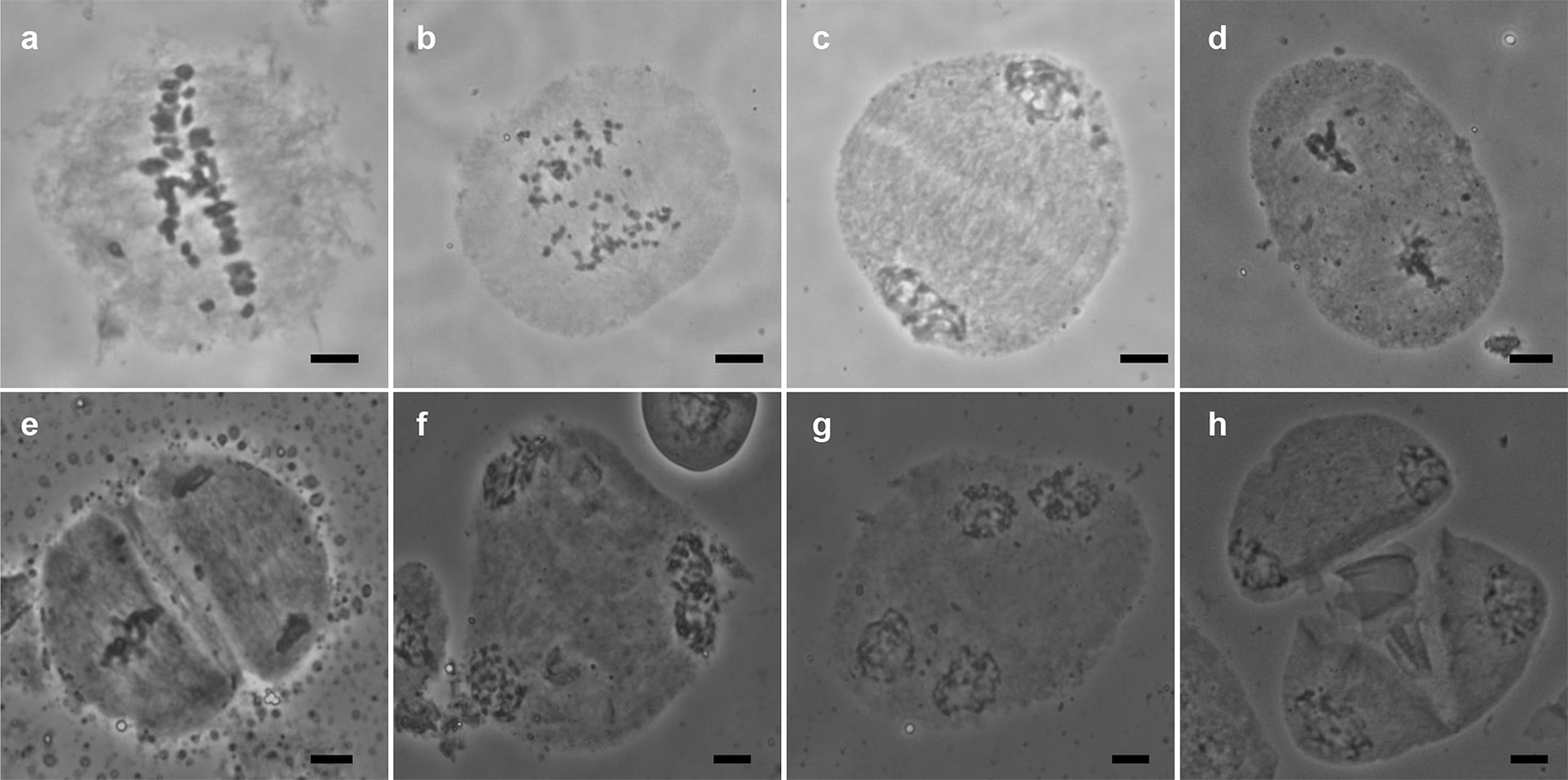

Supplement: Supplementary file 4 — Additional file 4: Fig. S4. Abnormal meiosis processes in F1 (Yacheng 96–66). a: Chromosome was not synchronized in metaphase I. b: Lagging chromosome in anaphase I. c: Lagging chromosome telophase I. d: No new cell plates formed in metaphase II. e and f: Asynchronous division in anaphase II. g: Cell with four nuclei. h: Asynchronous division in telophase II. Scale bars = 10 μm. [file 12870_2021_2911_MOESM4_ESM.tif]

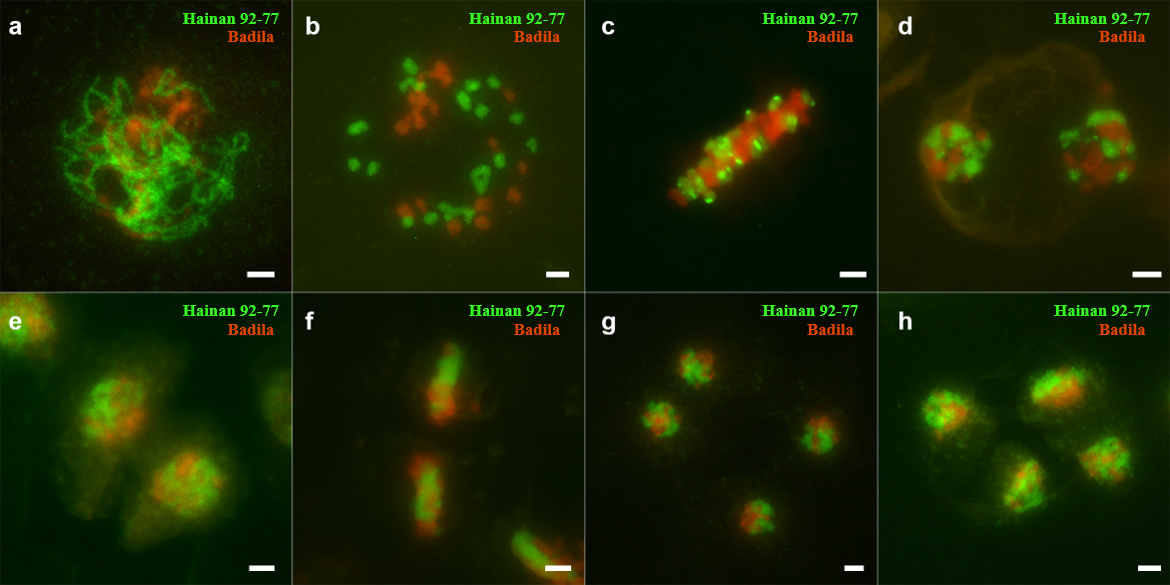

Supplement: Supplementary file 5 — Additional file 5: Fig. S5. GISH of F1 PMCs during normal meiosis. a: Pachytene. b: Diakinesis, white arrow refers to paired bivalents. c: Metaphase I. d: Anaphase I. e: Telophase I. f: Metaphase II. g: Anaphase II. h: Tetrad. Scale bars = 5 μm. [file 12870_2021_2911_MOESM5_ESM.tif]

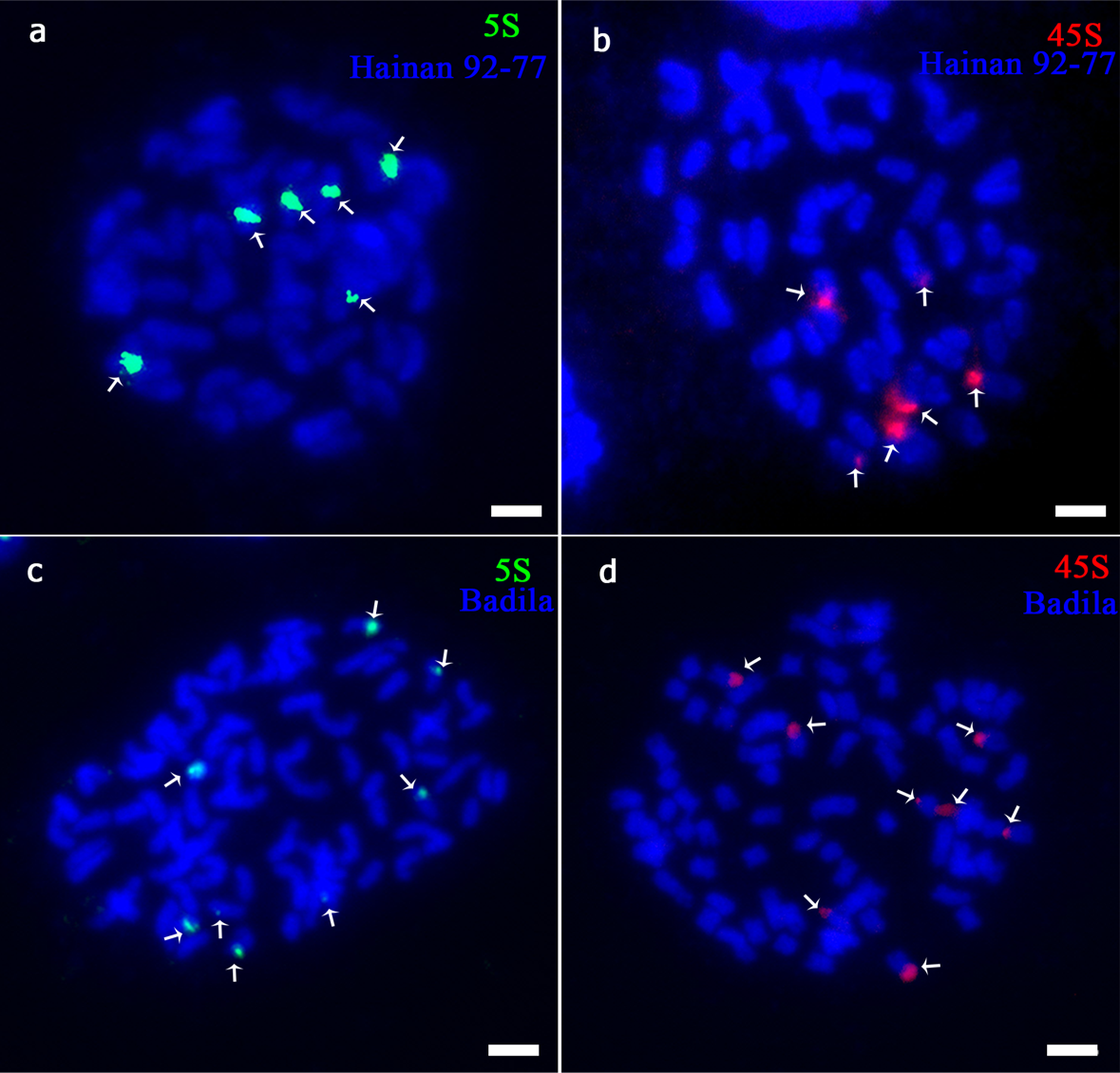

Supplement: Supplementary file 6 — Additional file 6: Fig. S. FISH mapping results for 45S rDNA and 5S rDNA at somatic cell chromosomes for Hainan 92–77 and Badila. a and b: somatic cell of Hainan 92–77. c and d: somatic cell of Badila. Arrows point to 45S rDNA and 5S rDNA foci. Scale bars = 5 μm. [file 12870_2021_2911_MOESM6_ESM.tif]

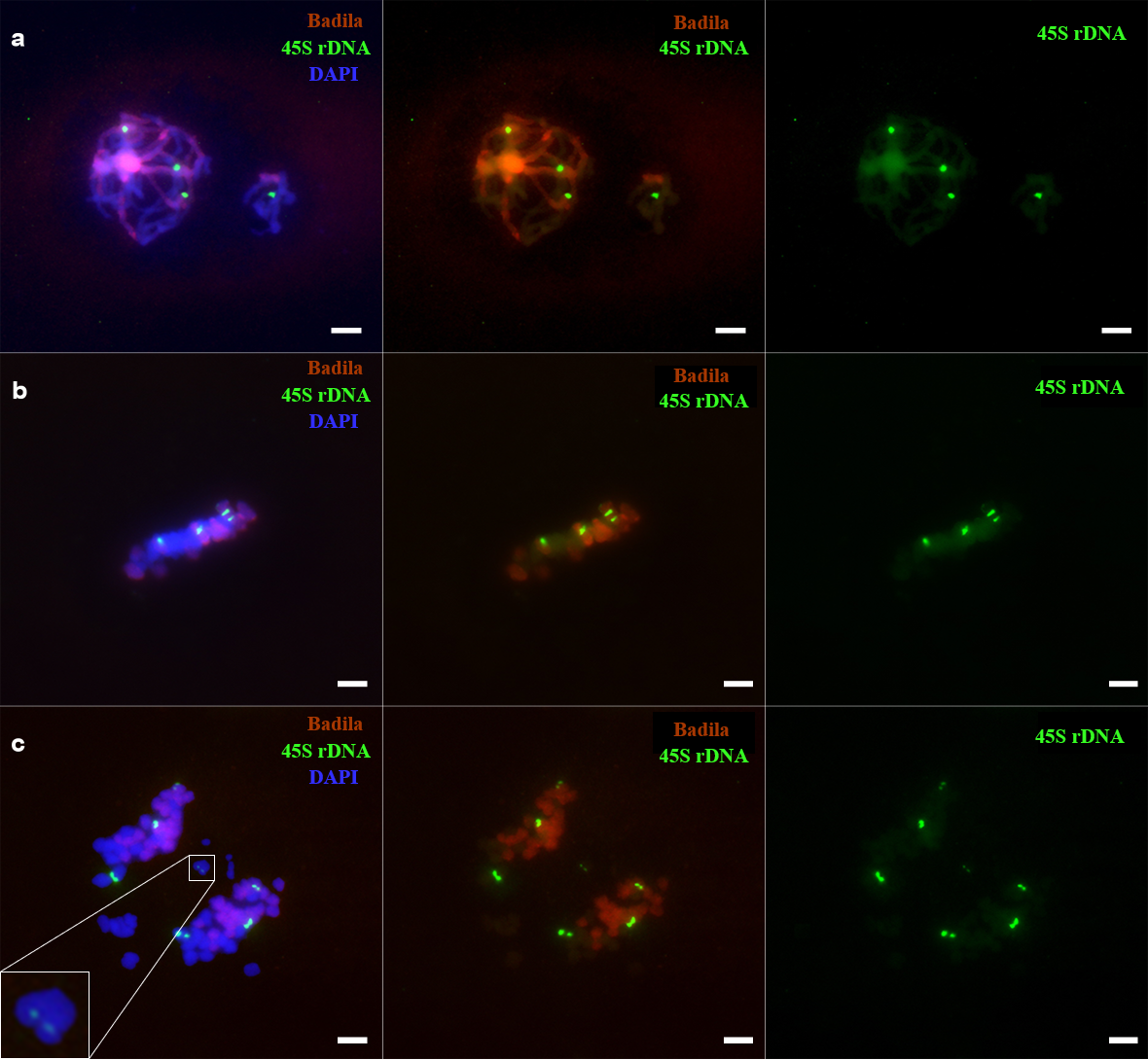

Supplement: Supplementary file 7 — Additional file 7: Fig. S7. Abnormal meiosis processes in F1 by 45S rDNA FISH mapping results. a: 45S site in pachytene. b: 45S site in metaphase I. c:45S site in anaphase I, the square indicated the lagging chromosome with 45S rDNA. Scale bars = 5 μm. [file 12870_2021_2911_MOESM7_ESM.tif]

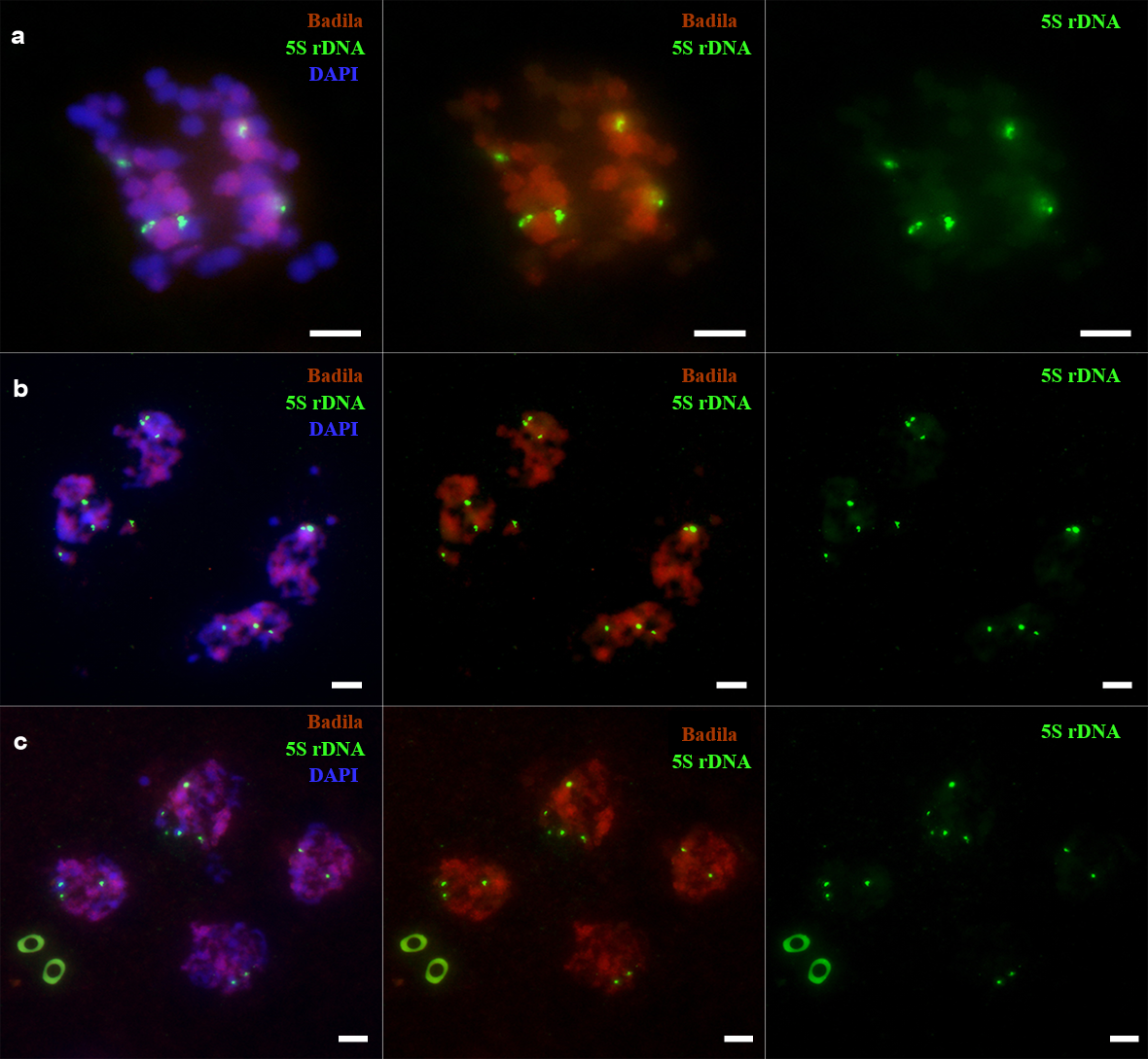

Supplement: Supplementary file 8 — Additional file 8: Fig. S8. Abnormal meiosis processes in F1 by 5S rDNA FISH mapping results. a: 5S site in anaphase I. b: 5S site in anaphase II. c: 5S rDNA site in tetrad. Scale bars = 5 μm. [file 12870_2021_2911_MOESM8_ESM.tif]

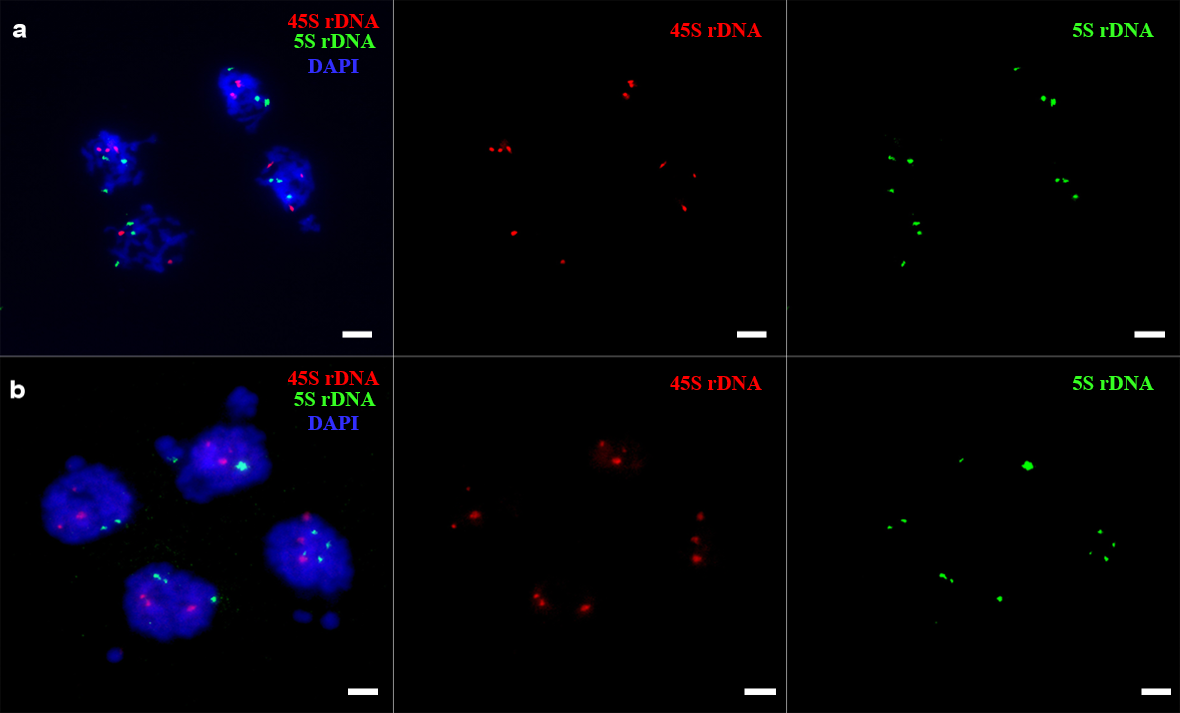

Supplement: Supplementary file 9 — Additional file 9: Fig. S9. Abnormal meiosis processes in F1 by 45S rDNA and 5S rDNA FISH mapping results. a and b: 45S rDNA and 5S rDNA site in tetrad. Scale bars = 5 μm. [file 12870_2021_2911_MOESM9_ESM.tif]
